# COVID-19 pandemic and the crude oil market risk: hedging options with non-energy financial innovations

**DOI:** 10.1186/s40854-021-00253-1

**Published:** 2021-05-10

**Authors:** Afees A. Salisu, Kingsley Obiora

**Affiliations:** 1 University of Ibadan Centre for Econometric and Allied Research, Ibadan, Oyo Nigeria; 2 Economic Policy Directorate, Central Bank of Nigeria, Abuja, Nigeria

**Keywords:** Pandemics, Financial innovations, Energy markets, Hedging, Optimal portfolio, I19, G15, G19, C52, G11

## Abstract

This study examines the hedging effectiveness of financial innovations against crude oil investment risks, both before and during the COVID-19 pandemic. We focus on the non-energy exchange traded funds (ETFs) as proxies for financial innovations given the potential positive correlation between energy variants and crude oil proxies. We employ a multivariate volatility modeling framework that accounts for important statistical features of the non-energy ETFs and oil price series in the computation of optimal weights and optimal hedging ratios. Results show evidence of hedging effectiveness for the financial innovations against oil market risks, with higher hedging performance observed during the pandemic. Overall, we show that sectoral financial innovations provide resilient investment options. Therefore, we propose that including the ETFs in an investment portfolio containing oil could improve risk-adjusted returns, especially in similar financial crisis as witnessed during the pandemic. In essence, our results are useful for investors in the global oil market seeking to maximize risk-adjusted returns when making investment decisions. Moreover, by exploring the role of structural breaks in the multivariate volatility framework, our attempts at establishing robustness for the results reveal that ignoring the same may lead to wrong conclusions about the hedging effectiveness.

## Introduction

This study seeks to unravel the hedging effectiveness of financial innovations in non-energy Exchange Traded Funds (ETFs) against oil price risks during COVID-19 pandemic. The research objective situates among numerous recent literature involving the connection between the current pandemic and the energy market (see, e.g., Apergis and Apergis 2020; Devpura and Narayan 2020a, b, c; Fu and Shen 2020; Gil-Alana and Monge 2020; Huang and Zeng 2020; Iyke 2020a; Liu et al. 2020; Narayan 2020a; Polemis and Soursou 2020; Prabheesh et al. 2020; Qin et al. 2020; Salisu and Adediran 2020). The widely held view is that the pandemic has impacted oil price negatively as lockdown measures at containing the virus have led to the shutdown of many companies. Meanwhile, the ensuing disruptions to global demand and supply chains have engendered irregular movements in energy prices (see also, Iyke and Ho 2020; Iyke 2020a). Although the motivation to hedge oil market risks is justified by studies suggesting the search for alternative hedging options for oil market risks (see Selmi et al. 2018; Olson et al. 2019; Sharma and Rodriguez 2019; Okorie and Lin 2020), the pandemic period offers yet greater motivation in this regard. This is because the crisis affecting the market becomes heightened with other markets (e.g., equities and currencies) that could be available to investors for diversification, which have also been impacted adversely by the pandemic(see Gil-Alana and Claudio-Quiroga 2020; Salisu et al. 2020a, b; Sharma 2020; Iyke 2020b; Narayan 2020b, c; Narayan et al. 2020).^1^

Therefore, this study contributes to the literature by exploring alternative hedging options for oil risks in financial innovations based on the ETFs, whose potential for hedging is increasingly gaining relevance in the literature. See arguments regarding the classes of financial innovations with low/negative correlations with most traditional portfolios and their potential risk-free nature qualifying them for hedging roles in Alexander and Barbosa (2008), Tari (2010), Agapova (2011), Gao (2012), Sharma and Rodriguez (2019), and Cheema et al. (2020). More specifically, many studies have discussed the strengths of ETFs as an important financial innovation and alternative investment assets (Agapova 2011; Gao 2012). More generally, financial innovations possess outstanding qualities; they are flexible investment options that offer risk-averse investors the prospect of holding a diversified basket of assets (although traded as single stocks as found in major global exchanges) without the need to trade in the physical assets defined in conventional investment portfolios (Dannhauser 2017; Marskz and Lechman 2018; Naeem et al. 2020; Ozdurak and Ulusoy 2020; Sakarya and Ekinci 2020).

**Fig. 1:**
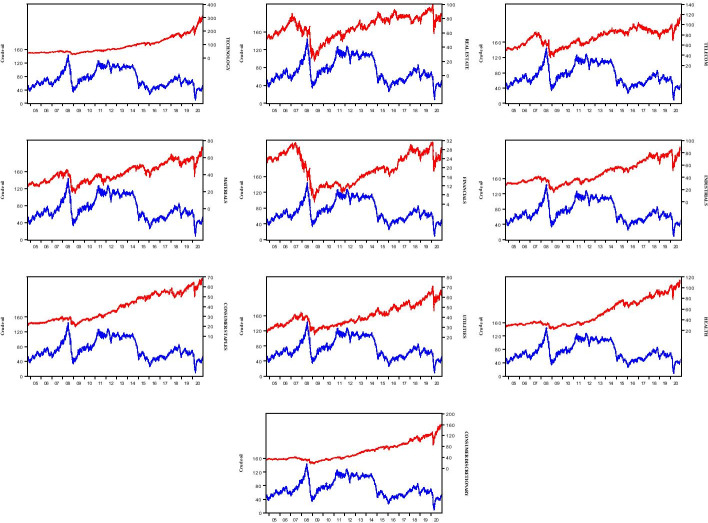
Pairwise graphs between non-energy sector ETFs and crude oil prices

We approach the contribution of the study by focusing on financial innovations in non-energy ETFs because we are interested in evaluating the hedging powers for oil price risk. Therefore, the energy components are isolated as the conventional wisdom in the literature; that is, investment assets in the same market/sector are believed to be positively correlated, and therefore, one cannot serve as a good hedge against another because both move in the same direction (see also, El-Sharif et al. 2005; Naeem et al. 2020; Ozdurak and Ulusoy 2020). For instance, Fig. 1 in the appendix depicts positive co-movements between energy sector financial innovations and the WTI oil price in 7 out of 10 sectors selected. Hence, the exclusion of energy sector’s financial innovations in the analysis of the hedging potential of financial innovations is justified. Thus, we consider 10 non-energy sectoral classifications of non-energy ETFs (see Table 1) as each of these financial innovations signifies a claim on similar underlying assets in the sectors (see Agapova 2011) and is expected to be negatively correlated with the oil market for possible risk hedging benefits.

**Table 1:** Non-energy exchange traded funds

Sector	ETF proxy	Symbol
Consumer discretionary	Consumer discretionary select sector SPDR fund	XLY
Consumer staples	Consumer staples select sector SPDR fund	XLP
Financials	financial select sector SPDR fund	XLF
Health	Health care select sector SPDR fund	XLV
Industrials	Industrial select sector SPDR fund	XLI
Materials	Materials select sector SPDR fund	XLB
Real estate	Vanguard real estate Index fund	VNQ
Technology	Invesco QQQ	QQQ
Telecom	Vanguard communication services ETF	VOX
Utilities	Utilities select sector SPDR fund	XLU

*Source*: www.etfdb.com/etfs/sector

The selected ETFs are based on Exchange Traded Funds categorization and ranking by the EFT database as at the end of December 2020

We employ the vector autoregressive moving average of the generalized autoregressive conditional heteroscedastic family (VARMA-GARCH) as the underlying model for the hedging relationship between oil price and non-energy financial innovations. This modeling framework becomes relevant after rounds of preliminary data testing including the graphical analysis showing largely negative co-movements between the variables and tests for serial correlation, conditional heteroscedasticity, sign-bias, and asymmetry, which all indicate the need to capture ARCH effects, asymmetry, and possible time-variation in the model (see also, Arouri and Nguyen 2010; Arouri et al. 2011a, b; Arouri et al. 2011a, b; Salisu and Mobolaji 2013; Salisu and Oloko 2015a; Salisu et al. 2020a, b, among others). In addition, the technique employed for the analysis tends to offer superior forecast performance relative to other competing models such as vector autoregressive (VAR-based) models and its variants (see Lypny and Powalla 1998; Lee et al. 2005; Yang and Lai 2009) in the modeling financial series with the foregoing statistical features thrown up at the pre-estimation stage.

To achieve the stated objective, we obtain the optimal hedge ratio (OHR) and optimal portfolio weight (OPW) associated with an investment in oil and non-energy financial innovations. Overall, we find that sectoral financial innovations are robust and resilient alternative investments. Further, we suggest that including them in an oil-based investment portfolio could provide alternative valuable asset class that can improve the risk-adjusted returns for investors, especially during a crisis. Therefore, when making investment decisions, investors in the global crude oil market that seek to maximize risk-adjusted returns are likely to find the results useful. For robustness, we test and account for structural breaks in the estimation process. The presence of the breaks shows that the optimal portfolio combination of financial innovations and oil could be over-estimated, whereas the hedging effectiveness could be underestimated when such breaks are ignored. In other words, ignoring any significant structural break, when in fact it exists, may lead to wrong conclusions about hedging effectiveness.

Following this background, we offer some preliminary analyses in “Data and methodology” section to determine the appropriate model for analyses. In “Analysis” section, we evaluate the relative hedging effectiveness of financial innovations for crude oil market risk due to the pandemic. In  “Robustness—accounting for structural breaks” section, we discuss the additional results for robustness, and in “Conclusion” section, we conclude the paper.

## Data and methodology

### Data description and summary statistics

The dataset used in the empirical estimation comprises daily prices of top-ranked non-energy ETFs^2^ and crude oil (using the West Texas Intermediate crude oil price as a proxy^3^) and covers the period between August 2004 and December 2020. The non-energy ETFs considered are Technology, Healthcare, Real estate, Materials, Consumer discretionary, Financials, Industrials, Utilities, Consumer staples and Telecom sectors (see Killa 2020).^4^ Table 1 highlights the selected ETFs for the 10 sectors (excluding the energy sector). Similarly, daily data on the sectoral ETF series are collected from *finance.yahoo.com*, and crude oil spot prices are obtainable from the US Energy Information Administration Database (https://eia.gov). To evaluate the impact of the unprecedented COVID-19 pandemic outbreak on the hedging relationship, we partition the full data sample (8/01/2004 to 12/30/2020) into pre-COVID (8/01/2004 to 12/31/2019) and COVID (1/2/2020 to 12/30/2020) periods.

Table 2 summarizes the statistics consisting of the mean, maximum, minimum, standard deviation, skewness, and kurtosis, of the return series of both the ETFs and oil prices. The mean values of the returns series for the 10 non-energy ETF sectors under consideration indicate positive average returns, both for the full and pre-COVID-19 sample periods. However, during the COVID sample, we find negative mean values for four sectors, namely, financials, industrials, real estate and technology sectors, whereas others remain positive. Meanwhile, the overall mean value involving the full sample for the oil sector is negative, whereas it is mixed for the two sub-sample. Moreover, it is positive for the pre-COVID-19 sample, whereas it is negative for the COVID-19 period. The standard deviation, which gives an insight into the volatility of the return series, reveals higher values during COVID-19 for the non-energy ETFs than the full sample and pre-COVID periods. This indicates that the ETFs exhibit more volatility during the COVID-19 period than the pre-COVID-19 sample. In addition, all the series are negatively skewed during COVID-19, given the negative values of the skewness statistics and are leptokurtic. Unsurprisingly, all the return series exhibit a conditional heteroscedasticity effect that must be dealt with in the estimation process required for the hedging analysis. A pairwise graphical representation between crude oil price and each non-energy sector ETFs shows evidence of opposite movements, which somewhat attests to the potential of the ETFs as a good hedge against oil price risk.

**Table 2:** Summary statistics for non-energy ETFs and oil returns

	Consumer discretionary	Consumer staples	Financials	Health	Industrials	Materials	Real estate	Technology	Telecom	Utilities	Oil
*Full data sample (8/01/2004 to 12/30/2020)*											
Mean	0.040	0.028	0.006	0.033	0.028	0.024	0.013	0.054	0.022	0.022	0.040
Maximum	12.316	11.534	25.090	10.244	10.061	11.207	19.487	9.542	26.304	12.367	12.316
Minimum	− 14.565	− 11.673	− 19.660	− 13.705	− 14.255	− 20.510	− 16.546	− 11.364	− 14.585	− 10.746	− 14.565
Std. Dev	1.462	1.000	2.019	1.150	1.414	1.582	1.897	1.329	1.399	1.194	1.462
Skewness	− 0.603	− 1.057	0.303	− 0.685	− 0.575	− 0.934	− 0.200	− 0.671	1.024	− 0.497	− 0.603
Kurtosis	17.637	24.383	22.990	15.522	13.682	17.431	20.136	10.602	45.652	16.146	17.637
*Before COVID-19 (8/01/2004 to 12/31/2019)*											
Mean	0.037	0.028	0.007	0.033	0.028	0.021	0.016	0.048	0.017	0.025	0.010
Maximum	0.087	0.040	0.054	0.083	0.071	0.073	0.068	0.131	0.045	0.060	0.036
Minimum	− 14.565	− 11.673	− 19.660	− 13.705	− 14.255	− 14.782	− 16.546	− 11.364	− 14.585	− 10.746	− 16.832
Std. Dev	1.403	0.952	1.969	1.107	1.332	1.496	1.868	1.262	1.359	1.112	2.150
Skewness	− 0.517	− 0.881	0.451	− 0.665	− 0.524	− 0.537	− 0.149	− 0.570	1.360	− 0.165	0.124
Kurtosis	18.880	26.295	25.529	17.004	13.953	11.765	21.854	11.048	52.910	15.618	7.807
*COVID-19 sample (1/2/2020 to 12/30/2020)*											
Mean	0.100	0.027	− 0.021	0.042	0.030	0.063	− 0.038	0.159	0.101	− 0.018	0.100
Maximum	8.923	5.148	8.774	4.795	8.319	10.899	7.942	6.055	6.238	6.594	8.923
Minimum	− 10.963	− 9.144	− 12.379	− 7.824	− 11.780	− 20.510	− 10.911	− 9.031	− 9.296	− 10.511	− 10.963
Std. Dev	2.181	1.567	2.679	1.676	2.338	2.571	2.314	2.104	1.911	2.081	2.181
Skewness	− 0.914	− 1.470	− 0.616	− 0.689	− 0.599	− 1.976	− 0.585	− 0.979	− 1.012	− 1.111	− 0.914
Kurtosis	8.782	11.278	6.967	6.572	7.403	20.422	6.547	5.831	6.926	9.015	8.782

### The model

This study employs the GARCH-based VARMA model proposed by Ling and McAleer (2003). The VARMA-GARCH models were featured as prominent instruments used in empirical literature for modeling interdependencies among financial time series with or without asymmetric shock effects (see Salisu and Mobolaji 2013; Salisu and Oloko 2015b; Al-Maadid et al. 2017; Salisu et al. 2020a, b). However, the choice of appropriate variants, that is, between constant conditional correlations (CCC) or its dynamic variant DCC, and between symmetric and asymmetric effects, is determined based on the outcomes of certain formal pretests.^5^ The general version of the VARMA-GARCH model has two parts: the mean equation part and the variance equation part. The former is typically a VAR model, and the latter is specified in a way that mimics the VARMA comprising ARCH and GARCH terms. Consequently, we construct a bivariate VARMA-GARCH(1,1) model and specify the mean equations that capture the return spillover effects between the two series under consideration, that is, ETF and crude oil price, and vice versa:,^6^,^7^

1 $$r_{t}^{oil} = \varphi^{oil} + \phi^{oil} r_{t - 1}^{oil} + \theta^{oil} r_{t - 1}^{etf} + \varepsilon_{t}^{oil}$$

2 $$r_{t}^{etf} = \varphi^{etf} + \phi^{etf} r_{t - 1}^{etf} + \theta^{etf} r_{t - 1}^{etf} + \varepsilon_{t}^{etf}$$

where $r_{t}^{etf}$ and $r_{t}^{oil}$ respectively denote each of non-energy sector’s ETFs and crude oil price return in period $t$; $\varphi^{etf}$ and $\varphi^{oil}$ are constant terms; $\phi^{etf}$ and $\phi^{oil}$ are coefficients of the lagged terms of own-returns respectively for non-energy ETF and crude oil; $\theta^{etf}$ and $\theta^{oil}$ are coefficients of the lagged terms of cross-return spillovers; and $\varepsilon_{t}^{etf}$ and $\varepsilon_{t}^{oil}$ are independently and identically distributed errors. Note that the superscripts, *oil* and *etf,* respectively, denote oil price and ETF returns. The conditional variance equations that provide the computation of the volatility spillover effects between the two asset classes are specified in Eqs. (3) and (4) for non-energy ETF and crude oil price returns, respectively:

3 $$h_{t}^{etf} = c^{etf} + \alpha_{1}^{etf} \left( {\varepsilon_{t - 1}^{etf} } \right)^{2} + \alpha_{2}^{etf} \left( {\varepsilon_{t - 1}^{oil} } \right)^{2} + \beta_{1}^{etf} \left( {h_{t - 1}^{etf} } \right) + \beta_{2}^{etf} \left( {h_{t - 1}^{oil} } \right)$$

4 $$h_{t}^{oil} = c^{oil} + \alpha_{a}^{oil} \left( {\varepsilon_{t - 1}^{oil} } \right)^{2} + \alpha_{b}^{oil} \left( {\varepsilon_{t - 1}^{etf} } \right)^{2} + \beta_{a}^{oil} \left( {h_{t - 1}^{oil} } \right) + \beta_{b}^{oil} \left( {h_{t - 1}^{etf} } \right)$$

These equations show that conditional variance for each sector depends on its immediate past values and innovations and the past values and innovations of the other sector. The parameters $\alpha_{i}$ and $\beta_{i}$ (where *i* = 1, 2) measure the shock and volatility spillover effects between the two return series, respectively, whereas the superscripts identify each series. Meanwhile, subscripts 1and 2, respectively, capture own- and cross-spillover effects. The conditional covariance, which is preliminarily assumed to be of CCC,^8^ is expressed as

5 $$h_{t}^{EO} = \rho^{EO} \times \sqrt {h_{t}^{etf} } \times \sqrt {h_{t}^{oil} }$$

where $\rho^{EO}$ is the conditional constant correlations between non-energy financial innovations and crude oil returns. In line with the objective of this paper, the estimated coefficients obtained from the VARMA-GARCH model are employed to evaluate the optimal weights and hedging effectiveness of non-energy sectoral financial innovations in an investment portfolio containing oil. The OPW establishes the proportion of investments in ETFs and crude oil to be included in a portfolio to ensure optimality. Significant volatility spillovers between two investment assets in a given portfolio may indicate that investments in the two assets are volatile and susceptible to risk and uncertainty. Hence, investors engage in hedging to mitigate such associated risks through investment in futures contract without jeopardizing expected future returns. Following the approach proposed by Kroner and Ng (1998) and Arouri et al. (2011a, b), we construct an OPW of holding the two assets (i.e., ETFs and crude oil) using the conditional variance and covariance estimates obtained after estimating Eqs. (3), (4), and (5):

6 $$\varpi_{EO,t} = \frac{{h_{t}^{etf} - h_{t}^{EO} }}{{h_{t}^{oil} - 2h_{t}^{EO} + h_{t}^{etf} }}$$

and,

7 $$\varpi_{EO,t} = \left\{ {\begin{array}{*{20}l} {0,} \hfill & {{\text{if }}\varpi_{EO,t} < 0} \hfill \\ {\varpi_{EO,t} ,} \hfill & {{\text{if 0 < }}\varpi_{EO,t} \le 1} \hfill \\ {1,} \hfill & {{\text{if }}\varpi_{EO,t} > 1} \hfill \\ \end{array} } \right.$$

where $\varpi_{EO,t}$ denotes the weight of non-energy sector’s ETFs in a one-dollar ETF/crude oil investment portfolio at time $t$. Also, the term—$h_{t}^{EO}$ is the conditional covariance between the ETF and crude oil returns at time $t$. Meanwhile, the OHR between each non-energy ETF and crude oil return is defined as

8 $$\alpha_{EO,t} = \frac{{h_{t}^{EO} }}{{h_{t}^{etf} }}$$

where $\alpha_{EO,t}$ is the OHR between the oil and each non-energy sector’s ETF under consideration. The description of the data used, including preliminary analyses and formal pretests, is discussed in the next section.

## Analysis

### Preliminary tests

We begin the results section with the formal preliminary tests conducted to determine the appropriate variant of the VARMA-GARCH model to be adopted for the main estimation, as discussed in the modeling section. The estimates obtained from the GARCH models are crucial in the estimation of the OPW and hedging effectiveness between each considered non-energy ETF and oil return. The considered pretests include serial correlation, conditional heteroscedasticity, asymmetry, and conditional correlation tests. The serial correlation test is conducted using Ljung-Box Q-statistics, whereas the ARCH-LM test is used for the conditional heteroscedasticity test over pre-determined lag lengths of 5 and 10. We test for asymmetry using Engle and Ng’s (1993) sign and bias tests, and we used Engle and Sheppard’s (2001) test to evaluate the presence or absence of the CCC in the multivariate volatility model. All the results of the pretests are summarized in Tables 3 and 4.

**Table 3:** Conditional Heteroscedasticity and Serial Correlation Tests

	Consumer discretionary	Consumer staples	Financials	Health	Industrials	Materials	Real estate	Technology	Telecom	Utilities	Oil
*Full data sample (8/01/2004 to 12/30/2020)*											
ARCH_5_	257.47***	253.95***	166.4***	239.88***	276.62***	170.62***	277.99***	211.84***	86.82***	316.41***	127.05***
ARCH_10_	184.49***	142.23***	98.37***	127.97***	147.75***	91.85***	157.70***	119.92***	49.25***	165.71***	80.83***
LB_5_	7.62	37.88***	17.94***	14.97***	3.68	27.76***	23.20***	5.47	24.60***	26.65***	11.09**
LB_10_	15.76*	46.66***	32.72***	18.55**	12.74	29.39***	43.42***	11.42	35.08***	39.67***	43.62***
LB^2^_5_	2351***	1811***	1323***	1367***	2183***	1433***	2241***	1803***	570.66***	1980***	575.91***
LB^2^_10_	4214***	2428***	2158***	1779***	3072***	1986***	3972***	2957***	738.57***	2838***	855.38***
*Before COVID-19 sample (8/01/2004 to 12/31/2019)*											
ARCH_5_	286.998***	283.988***	156.510***	227.041***	317.81***	310.75***	269.78***	210.64***	84.052***	290.55***	41.365***
ARCH_10_	191.811***	155.531***	93.627***	120.996***	168.55***	185.68***	152.33***	121.52***	47.308***	149.175***	29.142***
LB_5_	21.367***	32.243***	22.350***	20.211***	5.310	15.695***	27.894***	4.873	37.97***	27.832***	2.355
LB_10_	35.410***	39.054***	39.388***	26.509***	16.299*	18.746**	45.79***	10.969	53.91***	57.244***	13.04
LB^2^_5_	2422***	1946***	1239***	1275***	2455***	2674***	2175***	1795***	537.31***	1883***	291.47***
LB^2^_10_	4367***	2409***	2030***	1608***	3278***	4083***	3873***	2870***	680.39***	2440***	552.08***
*During COVID-19 Sample (1/2/2020 to 12/30/2020)*											
ARCH_5_	13.15***	8.43***	12.35***	15.58***	7.42***	3.84***	10.51***	8.74***	7.37***	23.85***	5.58***
ARCH_10_	7.58***	12.50***	7.08***	10.01***	5.45***	1.91**	6.31***	5.11***	4.98***	15.73***	3.63***
LB_5_	7.43	19.03***	13.31**	5.10	7.18	15.35***	5.40	3.14	7.94*	5.78	4.43
LB_10_	11.78	22.95***	19.21**	9.21	12.87	16.04*	9.99	4.86	17.53**	16.24*	17.90**
LB^2^_5_	77.37***	57.27***	83.71***	82.23***	50.24***	22.85***	65.71***	58.57***	51.48***	102.82***	26.82***
LB^2^_10_	119.01***	161.11***	128.39***	150.93***	98.50***	25.09***	109.91***	99.81***	96.03***	208/14***	39.89***

ARCH_5_ and ARCH_10_ indicate the ARCH LM tests at 5 and 10 lags respectively. The Ljung-Box tests—LB and LB^2^ test for autocorrelations and respectively utilize the standardized residuals in levels and squared standardized residuals. A non-rejection of the null hypotheses for the ARCH LM and Ljung-Box tests implies the absence of conditional heteroscedasticity and serial correlation respectively while a rejection implies otherwise. The superscripts ***, ** and * indicate statistical significance respectively at 1%, 5% and 10% levels

**Table 4:** Sign Bias and Asymmetry Tests

	Consumer discretionary	Consumer staples	Financials	Health	Industrials	Materials	Real estate	Technology	Telecom	Utilities	Oil
*Full data sample (8/01/2004 to 12/30/2020)*											
Sign bias	1.86*	0.84	1.20	2.25**	1.95*	2.14**	2.02**	2.11*	1.12	1.26	0.013
Negative bias	0.92	1.42	1.39	0.97	1.25	1.35	1.14	1.03	2.82***	1.14	2.79***
Positive bias	1.35	0.81	1.13	0.12	0.80	0.22	1.23	0.17	0.58	1.23	2.38**
Joint bias	16.85***	8.76**	10.98**	13.81***	17.40***	16.74***	17.00***	11.23**	19.99***	13.38***	21.04***
ES	13.93***	2.74	16.49***	13.49***	11.90***	9.87***	8.76**	14.25***	16.10***	6.30**	
*Before COVID-19 sample (8/01/2004 to 12/31/2019)*											
Sign bias	2.122**	0.879	1.187	1.799*	2.003*	2.070**	1.917*	2.614***	1.064	1.061	1.213
Negative bias	1.054	1.425	1.551	1.188	1.229	1.631	1.717*	1.459	2.980***	1.675*	0.830
Positive bias	1.127	0.170	1.083	0.056	0.587	0.156	0.602	1.047	0.391	0.429	1.819*
Joint bias	18.917***	6.595*	11.501***	11.42***	16.88**	18.60***	16.27***	31.53***	20.26***	11.36***	9.609**
ES	7.70**	1.735	5.772*	9.007**	12.16**	7.593**	7.53**	10.88***	9.302***	4.04	
*During COVID-19 sample (1/2/2020 to 12/30/2020)*											
Sign bias	0.40	1.59	0.41	1.67*	0.98	0.37	1.68*	1.49	1.70*	0.73	0.47
Negative bias	0.46	0.36	0.60	0.60	1.05	0.23	1.70*	0.85	0.91	0.58	1.55
Positive bias	0.57	0.85	0.36	0.42	0.31	0.71	1.09	0.02	0.13	1.38	0.44
Joint bias	0.89	7.26*	0.67	3.29	1.95	0.56	7.61*	3.30	3.61	4.59	4.35
ES	2.62	1.37	8.32**	5.22*	0.91	0.52	0.17	3.79	4.24	0.82	

ES test is the Engle and Sheppard (2001) CCC $\chi_{2}^{2}$ test; the values in parentheses denote the computed probability values. The superscripts ***, ** and * indicate statistical significance respectively at 1%, 5% and 10% levels.

The results of the ARCH-LM tests indicate that all returns exhibit conditional heteroscedasticity with the hypothesis of no ARCH effects rejected for the series under consideration. Therefore, such effects must be accommodated in the empirical estimation. The Ljung-Box tests, using both the correlogram Q-statistic and its squared variant, further confirm the presence of serial correlation across all return series, both at 5 and 10 lag orders. Table 4 summarizes the results of Engle and Ng’s (1993) sign and bias tests and Engle and Sheppard’s (2001) tests. The estimated results of Engle and Ng’s (1993) sign and joint size bias tests, both of which evaluate the evidence of asymmetric effects in the relationship between each ETF and oil price return, confirm the presence of the same nexus for the pre-covid sample. Meanwhile, the results show evidence of asymmetric relationship only for the financial innovations in Consumer Staples and Real Estate sectors. Finally, the results of Engle and Sheppard’s (2001) test provide statistically significant support for dynamic conditional correlations for almost all the sectors considered in the full sample and pre-COVID periods, whereas only two sectors, namely, Financial and Health, exhibit dynamic conditional correlations using the pandemic sample period.

### Main results^9^

Table 5 presents the results for the OPW and OHR used to evaluate the hedging capabilities of non-energy financial innovations for crude oil price risks, both before and after the emergence of the COVID-19 outbreak. This rests on the idea that the risks in taking a long position in a given asset (crude oil) can be offset by taking a short position in alternative assets (in this case, the sectoral financial innovations) (see Kumar 2014). Since the outbreak of the COVID-19 pandemic, the ETF ecosystem has demonstrated its robustness and resilience by continuing to provide investors with alternative portfolios and diversification buffers to absorb investment risks from highly volatile global market (see Jin et al. 2020; Xavier 2020). Both the OPW and OHR are obtained using the estimates of the conditional variance and covariance from the estimation of the main model.

**Table 5:** Optimal portfolio weights and hedge ratios

	Full sample		Before COVID-19		During COVID-19	
	OPW	OHR	OPW	OHR	OPW	OHR
Consumer discretionary	0.7613	− 0.0063	0.8546	0.0890	0.8113	0.1816
Consumer staples	0.8411	− 0.0302	0.8855	0.0561	0.9162	0.1310
Financials	0.8334	0.0410	0.8537	0.0924	0.8187	0.1484
Health	0.8822	0.0233	0.8867	0.0559	0.8671	0.1378
Industrials	0.8553	0.0092	0.8185	0.1149	0.7214	0.2438
Materials	0.7869	0.0794	0.8027	0.1510	0.7405	0.2184
Real estate	0.8309	0.0086	0.6522	0.0940	0.5427	0.3126
Technology	0.8935	0.0257	0.8274	0.0983	0.7310	0.0937
Telecom	0.9034	0.0221	0.7435	0.0966	0.8039	0.1023
Utilities	0.8505	0.0291	0.8144	0.0595	0.7571	0.1015

The table reports average optimal portfolio weights (OPW) and optimal hedge ratios (OHR) for non-energy ETFs in an oil investment portfolio

The estimated OPWs show positive portfolio weight coefficients for all variants of ETF–oil portfolio combination. Using the full sample, the estimated results show that ETFs for the three sectors comprising telecommunications, technologies, and health recorded the highest OPW at 0.9034, 0.8935, and 0.8822, respectively. Moreover, the OPW estimates suggest that the optimal proportion of portfolios in crude oil assets and investments in non-energy ETFs is about 90%, 89%, and 88% for the telecommunications, technologies, and health sectors,,respectively. Meanwhile, OPW estimates for the COVID-19 sample period show the highest OPW for the Consumer Staples and health sectors’ ETFs. One key highlight of the OPW results is the difference in hedging effectiveness between ETFs and oil price risk particularly during the current pandemic. This is expected because sectoral responses and resistance vary due to different economic conditions and political events that are capable of influencing each sector (see Salisu et al. 2019a, b, c; Chang et al. 2020).

In a similar vein, the OHRs in a financial innovation—oil asset portfolio combination for each non-energy sector—are also summarized in Table 5. The estimated OHR statistics also show mixed results across the different sectors over the three data samples. However, an interesting observation from the estimated results is that the obtained OHR values increased in the pandemic period than the full sample and pre-COVID-19 sample. This observation appears consistent across the findings for the 10 non-energy sectors considered. The increased hedge ratios during the pandemic suggest that risks associated with oil assets can be hedged by taking a short position in the non-energy financial innovations (ETFs). These findings show positive portfolio weight coefficients and higher OHR across the sectors in the pandemic period. They further corroborate the findings that financial innovations during crisis continue to demonstrate high resilience and robustness in terms of providing alternative portfolio options and diversification buffers capable of absorbing investment risks associated with the highly volatile crude oil market (see also, Naeem et al. 2020; Xavier 2020). This implies that financial innovations, that is, ETFs in the non-energy sectors, provide hedging effectiveness for oil assets. However, the same may not be concluded for the conventional portfolio investment in the physical non-energy sector assets, especially during periods of financial crisis epitomized by the pandemic. We therefore suggest that investors in the global crude oil market seeking to maximize their risk-adjusted returns should find the financial innovations in the non-energy sectors to be worthwhile portfolio options in dealing with the crude oil market risk. More especially, during future crisis, investors will find greater diversified portfolio investment in financial innovations in Consumer Staples sector to be worthwhile smart risk hedging decisions.

## Robustness—accounting for structural breaks

For robustness, we extend the multivariate volatility analysis by testing and accounting for structural breaks, where such exist, to enhance the precision of the model. A good amount of available empirical literature suggested and demonstrated the importance of accounting for structural breaks alongside controlling for volatility while dealing with high frequency financial series (see, e.g., Narayan and Liu 2011, 2015; Salisu and Adeleke 2016; Salisu et al. 2016). The effects of ignoring structural shifts in the data have affected the optimal weights, OHR, and hedge effectiveness (see previous pieces of evidence in Babikir et al. 2012; Mongi and Dhouha 2016). Furthermore, Babikir et al. (2012) suggested that GARCH processes stationarity assumption cause problems during periods where structural breaks are present, and this may render the GARCH assumptions invalid. Besides, failure to account for such breaks when they exist could lead to upward biases in the degree of persistence in estimated GARCH models. Hence, we explore the existence or non-existence of structural breaks in the series under investigation and account for the same in our estimated multivariate volatility models.

To account for structural breaks, we follow a three-step procedure. First, we determine the presence of structural breaks in each series using the conventional Augmented Dickey-Fuller (Narayan and Liu 2015) and GARCH-based unit root tests. The unit root test results yield the break date for each series; all are summarized in Table 6 for the three sample periods. The second step requires regressing each non-energy sector’s ETF return and crude oil return on dummy variables constructed for the identified break dummies, that is

$$r_{it} = \theta + \sum\limits_{j = 1}^{N} {\tau_{j} D_{jit} } + \upsilon_{it}$$

where $D_{j} = 1$ for each $j$, and zero otherwise, where $j$ is the number of breaks. In the third step, we determine the break-adjusted returns ($r_{it}^{d}$), which is estimated as $r_{it}^{d} = r_{it} - \sum\limits_{j = 1}^{N} {\hat{\tau }_{j} D_{jit} }$ or simply $r_{it}^{d} = \theta + \hat{\upsilon }_{it}$. The estimated break-adjusted returns ($r_{it}^{d}$) are thereafter used in the returns and volatility modeling, as discussed earlier in the model section.

**Table 6:** Unit root test results

	Consumer discretionary	Consumer staples	Financials	Health	Industrials	Materials	Real estate	Technology	Telecom	Utilities	Oil
*Full Sample (2004-09-30 to 2020-12-30)*											
ADF [*I*(0)]	− 69.645^a^	− 37.086^a^	− 72.413^a^	− 50.688^a^	− 68.015^a^	− 67.559^a^	− 70.154^a^	− 68.742^a^	− 50.469^a^	− 49.927^a^	− 64.852^a^
NL [*I*(0)]	− 65.028^a^	− 67.101^a^	− 66.313^a^	− 67.406^a^	− 63.880^a^	− 64.313^a^	− 62.681^a^	− 64.975^a^	− 65.925^a^	− 65.636^a^	− 60.392^a^
Break date	10/16/2008	11/05/2004	9/19/2008	10/14/2008	10/14/2008	10/14/2008	11/25/2008	8/24/2015	10/06/2004	11/05/2004	4/21/2020
Nobs	4068	4068	4068	4068	4068	4068	4068	4068	4068	4068	4068
*Pre-COVID Sample (2004-09-30 to 2020-12-31)*											
ADF [*I*(0)]	− 47.715^a^	− 41.698^a^	− 70.343^a^	− 49.830^a^	− 66.328^a^	− 63.672^a^	− 34.996^a^	− 65.861^a^	− 49.649^a^	− 49.311^a^	− 61.038^a^
NL [*I*(0)]	− 62.757^a^	− 65.801^a^	− 64.841^a^	− 65.953^a^	− 62.314^a^	− 62.595^a^	− 60.980^a^	− 62.613^a^	− 63.647^a^	− 64.349^a^	− 59.952^a^
Break date	10/16/2008	10/13/2008	9/19/2008	10/14/2008	10/14/2008	10/14/2008	11/25/2008	8/24/2015	9/19/2008	10/10/2008	01/02/2009
Nobs	3818	3818	3818	3818	3818	3818	3818	3818	3818	3818	3818
*COVID Sample (2020-01-01 to 2020-12-30)*											
ADF [*I*(0)]	− 17.600^a^	− 16.857^a^	− 17.561^a^	− 16.594^a^	− 16.264^a^	− 19.295^a^	− 14.340^a^	− 17.992^a^	− 18.396^a^	− 14.839^a^	− 16.653^a^
NL [*I*(0)]	− 17.422^a^	− 15.681^a^	− 15.770^a^	− 14.541^a^	− 14.466^a^	− 14.188^a^	− 15.218^a^	− 17.667^a^	− 18.387^a^	− 14.463^a^	− 16.495^a^
Break date	04/07/2020	3/23/2020	03/12/2020	03/12/2020	03/12/2020	3/16/2020	3/17/2020	3/12/2020	03/12/2020	3/23/2020	4/21/2020
Nobs	249	249	249	249	249	249	249	249	249	249	249

ADF is the Augmented Dickey Fuller unit root test; NL is the GARCH-based unit root test with structural breaks proposed by Narayan and Liu (2015) and it is considered an alternative to the Narayan and Popp (2010) test due to the data frequency used in this study (see also Salisu and Adeleke 2016). Both unit root tests are conducted with a constant and a time trend. For want of space here, we use the superscripts a, b and c to denote statistical significance at 1%, 5% and 10% levels, respectively

Table 7 summarizes the estimated OPW and OHR using the structural breaks adjusted return series.^10^ The results show that accounting for the significant structural breaks in the ETFs and oil return series has implications on the optimal weights and OHR and, by extension, the hedging effectiveness for the considered assets portfolio combination. For instance, the estimated OPW coefficients seem to be over-estimated when structural breaks are ignored. This is valid across all sectors under consideration. Meanwhile, the overall estimated OHR coefficients increase after accounting for breaks. In other words, these results seem to imply that the hedging effectiveness of the financial innovations for oil investment risks is underestimated when significant structural breaks exist but are not accounted for (see also Mongi and Dhouha 2016). On the whole, ignoring any significant structural break, when in fact it exists, may lead to wrong conclusions about the hedging effectiveness.

**Table 7:** Optimal portfolio weights and hedge ratios using breaks adjusted series

	Full sample		Before COVID-19		During COVID-19	
	OPW	OHR	OPW	OHR	OPW	OHR
Consumer discretionary	0.8523	0.0921	0.8077	0.0946	0.8475	0.1047
Consumer staples	0.8961	0.0560	0.9038	0.0501	0.8990	0.0716
Financials	0.9163	0.1095	0.8628	0.0836	0.8534	0.1136
Health	0.9447	0.1074	0.9026	0.0477	0.8824	0.0715
Industrials	0.8681	0.1759	0.9118	0.0806	0.8059	0.1401
Materials	0.8655	0.1445	0.8479	0.1344	0.7996	0.1771
Real estate	0.7182	0.2311	0.8629	0.0492	0.6494	0.2698
Technology	0.8268	0.0525	0.9241	0.0697	0.6763	0.1507
Telecom	0.8650	0.0576	0.9308	0.0554	0.7845	0.1553
Utilities	0.8473	0.0774	0.8663	0.0478	0.7761	0.1736

The table reports average optimal portfolio weights (OPW) and optimal hedge ratios (OHR) for non-energy ETFs in an oil investment portfolio after adjusting for structural breaks in each of their return series

## Conclusion

This study investigates whether financial innovations in non-energy sectors that allow investors to trade in diversified portfolios of passive investments in these sectors could provide effective hedging alternatives for the global crude oil market investors. This becomes justified, especially, despite the recent pandemic with adverse effects on the energy and other conventional financial markets. We use the largest and top-performing ETFs from the 10 non-energy sectors as proxies of financial innovations to estimate the OPW and OHR, which are used to evaluate the hedging effectiveness in an investment portfolio that combines non-energy financial innovations and crude oil. The portfolio weights and hedge ratios are computed using the estimated conditional variance and covariance obtained from appropriate versions of the VARMA-GARCH models as informed by standard preliminary tests. In addition, we account for the impact of COVID-19 by classifying the data sample into two sub-samples—pre-COVID-19 samples and COVID-19 sample.

These findings support evidence of hedging effectiveness between considered sectoral financial innovations and oil price returns. Further, we report improved hedging performance during the pandemic, thus substantiating the earlier advancement for the consideration of sectoral financial innovations as resilient alternative investment options that could help improve the risk-adjusted returns for oil investors during a crisis. By further accounting for structural breaks in the analysis, we establish that the optimal portfolio combination of financial innovations and oil could be over-estimated, whereas the hedging effectiveness could be underestimated when such breaks are ignored. In other words, ignoring any significant structural break despite its existence may lead to wrong conclusions about the hedging effectiveness. Overall, investors in the global crude oil market that seek to maximize risk-adjusted returns should find the outcome of the study useful when making investment decisions.

Several possibilities exist for future researchers to extend this study. One of the immediate choices is to explore the hedging effectiveness of other forms of financial innovations excluding ETFs, such as Sukuk (Islamic) bonds, hedge funds, and mutual funds, for covering the oil market risks. In addition, other extensions like the expanded energy market risks can be explored in future studies.

## Figures and Tables

**Fig. 2:**
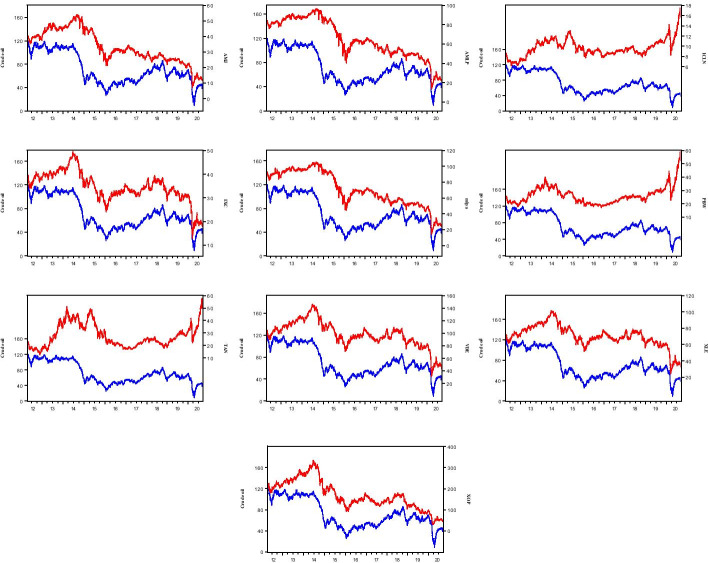
Co-movements between energy sector ETFs and crude oil prices

## Data Availability

The data that support the findings of this study are available on request from the corresponding author. Some of the data are not publicly available due to privacy or ethical restrictions.

## Appendix

See Fig. 2.

## Footnotes

1 For instance, on April 20, 2020, the West Texas Intermediate (WTI) dropped by a record 300% low (Devpura and Narayan 2020a, b, c). In the first quarter of 2020, global stock price recorded a loss of about 12.35% (Qin et al. 2020; Salisu et al. 2020a, b).

2 https://etfdb.com/etfs/sector/.

3 The West Texas Intermediate crude oil price is considered a good reflection of the global crude oil price (see Narayan and Gupta 2015).

4 https://finance.yahoo.com/news/top-ranked-etfs-stocks-top-150003045.html.

5 The preliminary test results are presented and discussed in the next section.

6 A similar methodology was recently adopted by Salisu, Vo and Lawal (2020a, b) to assess the hedging potential of gold against oil price risk.

7 We acknowledge that the interplay of several factors is responsible for the movements in global crude oil prices (some of which have been evaluated in other literature). However, during the outbreak of the coronavirus pandemic, there seems to be a consensus in the literature (see, e.g., Gil-Alana and Monge 2020; Narayan 2020a, b, c; Salisu, Ebuh, and Usman 2020a, b) that the huge decline in oil prices was mainly due to political and economic decisions meant to curtail the viral spread, such as economic lockdown and domestic and international travel restrictions. In addition, crude oil price has never recorded a negative price in its entire history until this period. Hence, the high impact of the pandemic might have overshadowed all other impacts. Notwithstanding, the way the VARMA-GARCH is specified accommodates shocks due to other factors that may be responsible for the unprecedented movements in oil prices. As the term implies, VARMA is a vector autoregressive moving average, which forms the components of the multivariate GARCH model used in this study.

8 An alternative variant of the variance equations is the one that allows for time variation in the conditional correlations, which is described as dynamic conditional correlations. To determine the choice of conditional correlations to account for the hedging analysis, we employ Engle and Sheppard’s (2001) test as part of the preliminary tests.

9 Given our objective of evaluating the hedging effectiveness between ETFs and crude oil returns, we suppress the results for both the conditional mean and variance equations of the VARMA-GARCH models including the post-estimation diagnostics that establish the goodness-of-fit and appropriateness of the models. These results are available and will be provided upon request.

10 The relevant preliminary diagnostics, including the conditional heteroscedasticity, autocorrelation, sign bias, and asymmetry tests, are conducted to determine the appropriate version of the multivariate volatility analysis across each non-energy financial innovation sector. The results and the multivariate volatility estimation results are not presented for space limitations but are available upon request.

## References

[CR1] Agapova A (2011). Conventional mutual index funds versus exchange-traded funds. J Financ Mark.

[CR2] Alexander C, Barbosa A (2008). Hedging index exchange traded funds. J Bank Finance.

[CR3] Al-Maadid A, Caporale GM, Spagnolo F, Spagnolo N (2017). Spillovers between food and energy prices and structural breaks. Int Econ.

[CR4] Apergis E, Apergis N (2020). Can the COVID-19 pandemic and oil prices drive the US Partisan Conflict Index?. Energy Res Lett.

[CR5] Arouri MEH, Nguyen DK (2010). Oil prices, stock markets and portfolio investment: evidence from sector analysis in Europe over the last decade. Energy Policy.

[CR6] Arouri MEH, Jouini J, Nguyen DK (2011). Volatility spillovers between oil prices and stock sector returns: implications for portfolio management. J Int Money Financ.

[CR7] Arouri MEH, Lahiani A, Nguyen DK (2011). Return and volatility transmission between world oil prices and stock markets of the GCC countries. Econ Model.

[CR8] Asness CS, Krail RJ, Liew JM (2001). Do hedge funds hedge?. J Portfolio Manag.

[CR9] Babikir A, Gupta R, Mwabutwa C, Owusu-Sekyere E (2012). Structural breaks and GARCH models of stock return volatility: the case of South Africa. Econ Model.

[CR10] Baur DG, Lucey BM (2010). Is gold a hedge or a safe haven? An analysis of stocks, bonds and gold. Financ Rev.

[CR11] Beck T, Demirgüç-Kunt A, Merrouche O (2010). Islamic vs. conventional banking: business model, efficiency and stability.

[CR12] Beck T, Chen T, Lin C, Song FM (2016). Financial innovation: the bright and the dark sides. J Bank Finance.

[CR13] Berg E, Schmitz B, Starp M (2006) Weather derivatives as an instrument to hedge against the risk of high energy cost in greenhouse production (No. 1004-2016-78626)

[CR14] Chang, B. H., Sharif, A., Aman, A., Suki, N. M., Salman, A., & Khan, S. A. R. (2020). The asymmetric effects of oil price on sectoral Islamic stocks: New evidence from quantile-on-quantile regression approach. *Resources Policy*, *65*, 101571.

[CR15] Chapra MU (2011) The global financial crisis: can islamic finance help? Islamic economics and finance. Springer, pp 135–142. 10.1057/9780230361133_5

[CR16] Cheema MA, Faff RW, Szulczuk K (2020). The 2008 global financial crisis and COVID-19 pandemic: How safe are the safe haven assets?. Covid Econ Vetted Real-Time Pap.

[CR17] Chen Z (1995). Financial innovation and arbitrage pricing in frictional economies. J Econ Theory.

[CR18] Chou YK (2007). Modeling financial innovation and economic growth: Why the financial sector matters to the real economy. J Econ Educ.

[CR19] Conlon T, McGee R (2020). Safe haven or risky hazard? Bitcoing during the COVID-19 bear market. Financ Res Lett.

[CR20] Corbet S, Larkin C, Lucey B (2020). The contagion effects of the COVID-19 pandemic: evidence from gold and cryptocurrencies. Financ Res Lett.

[CR21] Dannhauser CD (2017). The impact of innovation: Evidence from corporate bond exchange-traded funds (ETFs). J Financ Econ.

[CR22] Devpura N, Narayan PK (2020). Hourly oil price volatility: the role of COVID-19. Energy Res Lett.

[CR23] El-Sharif I, Brown D, Burton B, Nixon B, Russell A (2005). Evidence on the nature and extent of the relationship between oil prices and equity values in the UK. Energy Econ.

[CR24] Engle RF, Ng VK (1993). Measuring and testing the impact of news on. J Finance.

[CR25] Engle RF, Sheppard K (2001) Theoretical and empirical properties of dynamic conditional correlation MVGARCH. UCSD working paper No. 2001-15

[CR26] Fu M, Shen H (2020). COVID-19 and corporate performance in the energy industry. Energy Res Lett.

[CR27] Gao Y (2012) Hedging effectiveness of energy exchange traded funds. Doctoral dissertation, Concordia University

[CR28] Gil-Alana LA, Claudio-Quiroga G (2020). The COVID-19 impact on the asian stock markets. Asian Econ Lett.

[CR29] Gil-Alana LA, Monge M (2020). Crude oil prices and COVID-19: persistence of the shock. Energy Res Lett.

[CR30] Godil DI, Sarwat S, Sharif A, Jermsittiparsert K (2020). How oil prices, gold prices, uncertainty and risk impact Islamic and conventional stocks? Empirical evidence from QARDL technique. Resour Policy.

[CR31] Hasan MM, Dridi J (2010) The effects of the global crisis on Islamic and conventional banks: a comparative study. IMF working papers, pp 1–46. 10.1142/S1793993311000270

[CR32] Huang W, Zheng Y (2020) COVID-19: structural changes in the relationship between investor sentiment and crude oil futures price. Energy Res Lett 1(2)

[CR33] Investopedia (2020) Top health care stocks for July, 2020. Available online: https://www.investopedia.com/investing/top-healthcare-stocks/. Assessed 7 Nov 2020

[CR34] Iyke BN (2020). COVID-19: the reaction of US oil and gas producers to the pandemic. Energy Res Lett.

[CR35] Iyke BN (2020). The disease outbreak channel of exchange rate return predictability: evidence from COVID-19. Emerg Mark Financ Trade.

[CR36] Iyke BN (2020). Economic policy uncertainty in times of COVID-19 pandemic. Asian Econ Lett.

[CR37] Iyke BN, Ho SY (2020). Consumption and exchange rate uncertainty: evidence from selected Asian countries. World Econ.

[CR38] Jin J, Han L, Wu L, Zeng H (2020). The hedging effectiveness of global sectors in emerging and developed stock markets. Int Rev Econ Financ.

[CR39] Killa S (2020) Top-ranked ETFs, stocks from top sector of the last decade. Retrieved from https://finance.yahoo.com/news/top-ranked-etfs-stocks-top-150003045.html

[CR40] Kroner KF, Ng VK (1998). Modeling asymmetric comovements of asset returns. Rev Financ Stud.

[CR41] Kumar D (2014). Return and volatility transmission between gold and stock sectors: application of portfolio management and hedging effectiveness. IIMB Manag Rev.

[CR42] Lee M, Chiou J-S, Wu P-S, Chen C-D (2005). Hedging with S&P500 and E-mini S&P500 stock index futures. J Stat Manag Syst.

[CR43] Liang B (2001). Hedge fund performance: 1990–1999. Financ Anal J.

[CR44] Ling S, McAleer M (2003). Asymptotic theory for a vector ARMA–GARCH model. Econ Theory.

[CR45] Liu L, Wang E-Z, Lee C-C (2020). Impact of the COVID-19 pandemic on the crude oil and stock markets in the US: a time-varying analysis. Energy Res Lett.

[CR46] Lypny G, Powalla M (1998). The hedging effectiveness of DAX futures. Eur J Finance.

[CR47] Marszk A, Lechman E (2018). Tracing financial innovation diffusion and substitution trajectories. Recent evidence on exchange-traded funds in Japan and South Korea. Technol Forecast Soc Chang.

[CR48] Mishra S, Sharif A, Khuntia S, Meo SA, Khan SAR (2019). Does oil prices impede Islamic stock indices? Fresh insights from wavelet-based quantile-on-quantile approach. Resour Pol.

[CR49] Mohanty SK, Nandha M, Turkistani AQ, Alaitani MY (2011). Oil price movements and stock market returns: evidence from Gulf Cooperation Council (GCC) countries. Global Finance J.

[CR50] Mongi A, Dhouha HA (2016). Do structural breaks affect portfolio designs and hedging strategies? International evidence from stock-commodity markets linkages. Int J Econ Financ Issues.

[CR51] Naeem M, Umar Z, Ahmed S, Ferrouhi EM (2020). Dynamic dependence between ETFs and crude oil prices by using EGARCH-Copula approach. Phys A.

[CR52] Narayan S (2013). Foreign exchange markets and oil prices in Asia. J Asian Econ.

[CR53] Narayan PK (2020). Oil price news and COVID-19-Is there any connection?. Energy Res Lett.

[CR54] Narayan PK (2020). Has COVID-19 changed exchange rate resistance to shocks?. Asian Econ Lett.

[CR55] Narayan PK (2020). Did bubble activity intensify during COVID-19?. Asian Econ Lett.

[CR56] Narayan PK, Gupta R (2015). Has oil price predicted stock returns for over a century?. Energy Econ..

[CR57] Narayan PK, Liu R (2011). Are shocks to commodity prices persistent?. Appl Energy.

[CR58] Narayan PK, Liu R (2015). A unit root model for trending time-series energy variables. Energy Econ.

[CR59] Narayan PK, Phan DHB (2017). Momentum strategies for Islamic stocks. Pac Basin Finance J.

[CR60] Narayan PK, Popp S (2010). A new unit root test with two structural breaks in level and slope at unknown time. J Appl Stat.

[CR61] Narayan PK, Narayan S, Prasad A (2008). Understanding the oil price-exchange rate nexus for the Fiji islands. Energy Econ.

[CR62] Narayan PK, Phan DHB, Sharma SS (2019). Does Islamic stock sensitivity to oil prices have economic significance?. Pac. Basin Finance j..

[CR63] Narayan PK, Devpura N, Wang H (2020). Japanese currency and stock market—What happened during the COVID-19 pandemic?. Econ Anal Policy.

[CR64] Okorie DI, Lin B (2020). Crude oil price and cryptocurrencies: evidence of volatility connectedness and hedging strategy. Energy Econ.

[CR65] Olson E, Vivian A, Wohar ME (2019). What is a better cross-hedge for energy: equities or other commodities?. Global Finance J.

[CR66] Ozdurak C, Ulusoy V (2020). Price discovery in crude oil markets: intraday volatility interactions between crude oil futures and energy exchange traded funds. Int J Energy Econ Policy.

[CR67] Partnoy F, Thomas RS (2007) Gap filling, hedge funds, and financial innovation 6–21

[CR68] Polemis M, Soursou S (2020) Assessing the impact of the COVID-19 pandemic on the Greek energy firms: an event study analysis. Energy Res Lett 1(3)

[CR69] Prabheesh KP (2020). Dynamics of foreign portfolio investment and stock market returns during the COVID-19 pandemic: evidence from India. Asian Econ Lett.

[CR70] Prabheesh KP, Padhan R, Garg B (2020). COVID-19 and the oil price-stock market nexus: evidence from net oil-importing countries. Energy Res Lett.

[CR71] Qin M, Zhang Y-C, Su C-W (2020). The essential role of pandemics: a fresh insight into the oil market. Energy Res Lett.

[CR72] Rahim AM, Masih M (2016). Portfolio diversification benefits of Islamic investors with their major trading partners: evidence from Malaysia based on MGARCH-DCC and wavelet approaches. Econ Modell.

[CR73] Rizvi SAR, Arshad S, Alam N (2015). Crises and contagion in Asia Pacific—Islamic v/s conventional markets. Pac Basin Finance J.

[CR74] Sakarya B, Ekinci A (2020). Exchange-traded funds and FX volatility: evidence from Turkey. Central Bank Rev.

[CR75] Salisu A, Adediran I (2020). Uncertainty due to infectious diseases and energy market volatility. Energy Res Lett.

[CR76] Salisu AA, Adeleke AI (2016). Further application of Narayan and Liu (2015) unit root model for trending time series. Econ Modell.

[CR77] Salisu AA, Mobolaji H (2013). Modeling returns and volatility transmission between oil price and US–Nigeria exchange rate. Energy Econ.

[CR78] Salisu AA, Oloko TF (2015). Modeling oil price–US stock nexus: a VARMA–BEKK–AGARCH approach. Energy Econ.

[CR79] Salisu AA, Oloko TF (2015). Modelling spillovers between stock market and FX market: evidence for Nigeria. J Afr Bus.

[CR80] Salisu AA, Sikiru AA (2020). Pandemics and the Asia-Pacific Islamic stocks. Asian Econ Lett.

[CR81] Salisu AA, Ndako UB, Oloko TF, Akanni LO (2016). Unit root modeling for trending stock market series. Borsa Istanbul Rev.

[CR82] Salisu AA, Adekunle W, Alimi WA, Emmanuel Z (2019). Predicting exchange rate with commodity prices: New evidence from Westerlund and Narayan (2015) estimator with structural breaks and asymmetries. Resour Policy.

[CR83] Salisu AA, Raheem ID, Ndako UB (2019). A sectoral analysis of asymmetric nexus between oil price and stock returns. Int Rev Econ Finance.

[CR84] Salisu AA, Swaray R, Oloko TF (2019). Improving predictability of oil-US stock nexus: the role of macroeconomic variables. Econ Model.

[CR85] Salisu AA, Ebuh GU, Usman N (2020). Revisiting oil-stock nexus during COVID-19 pandemic: some preliminary results. Int Rev Econ Financ.

[CR86] Salisu AA, Vo XV, Lawal A (2020). Hedging oil price risk with gold during COVID-19 pandemic. Resour Policy.

[CR87] Selmi R, Mensi W, Hammoudeh S, Bouoiyour J (2018). Is Bitcoin a hedge, a safe haven or a diversifier for oil price movements? A comparison with gold. Energy Econ.

[CR88] Sharma SS (2020). A note on the asian market volatility during the COVID-19 pandemic. Asian Econ Lett.

[CR89] Sharma S, Rodriguez I (2019). The diminishing hedging role of crude oil: evidence from time varying financialization. J Multinatl Financ Manag.

[CR90] Smyth R, Narayan PK (2018). What do we know about oil prices and stock returns?. Int Rev Financ Anal.

[CR91] Statista (2020) How Covid 19 has impacted the global start up. Available online: https://www.statista.com/chart/22134/coronavirus-impact-on-startups/. Assessed 7 Aug 2020, pp 237–256

[CR92] Swaray R, Salisu AA (2018). A firm-level analysis of the upstream-downstream dichotomy in the oil-stock nexus. Global Finance J.

[CR93] Tari MJ (2010). Exchange‐traded funds (ETFs). In: Encyclopedia of quantitative finance, R. Cont (Ed.). 10.1002/9780470061602.eqf07035

[CR94] Tisdell CA (2020). Economic, social and political issues raised by the COVID-19 pandemic. Econ Anal Policy.

[CR95] Xavier J (2020) ETFs: passing the Covid-19 stress test | ETF Strategy. Retrieved October 11, 2020, from https://www.etfstrategy.com/etfs-passing-the-covid-19-stress-test-98547/

[CR96] Yang MJ, Lai YC (2009). An out-of-sample comparative analysis of hedging performance of stock index futures: dynamic versus static hedging. Appl Financ Econ.

[CR97] Yang CC, Brockett PL, Wen MM (2009) Basis risk and hedging efficiency of weather derivatives. J Risk Finance

[CR98] Zhang YJ, Wu YB (2019). The time-varying spillover effect between WTI crude oil futures returns and hedge funds. Int Rev Econ Financ.

